# *PAM* variants in patients with thyrotrophinomas, cyclical Cushing’s disease and prolactinomas

**DOI:** 10.3389/fendo.2023.1305606

**Published:** 2023-11-23

**Authors:** Sunita M. C. De Sousa, Angeline Shen, Christopher J. Yates, Roderick Clifton-Bligh, Stephen Santoreneos, James King, John Toubia, Giampaolo Trivellin, Andrea G. Lania, Constantine A. Stratakis, David J. Torpy, Hamish S. Scott

**Affiliations:** ^1^ Endocrine & Metabolic Unit, Royal Adelaide Hospital, Adelaide, SA, Australia; ^2^ South Australian Adult Genetics Unit, Royal Adelaide Hospital, Adelaide, SA, Australia; ^3^ Adelaide Medical School, University of Adelaide, Adelaide, SA, Australia; ^4^ Department of Diabetes and Endocrinology, Royal Melbourne Hospital, Melbourne, VIC, Australia; ^5^ Department of Medicine, University of Melbourne, Melbourne, VIC, Australia; ^6^ Cancer Genetics Laboratory, Kolling Institute, Royal North Shore Hospital, Sydney, NSW, Australia; ^7^ Sydney Medical School, The University of Sydney, Sydney, NSW, Australia; ^8^ Department of Endocrinology, Royal North Shore Hospital, Sydney, NSW, Australia; ^9^ Department of Neurosurgery, Royal Adelaide Hospital, Adelaide, SA, Australia; ^10^ Department of Surgery, University of Melbourne, Melbourne, VIC, Australia; ^11^ ACRF Cancer Genomics Facility, Centre for Cancer Biology, University of South Australia and SA Pathology, Adelaide, SA, Australia; ^12^ Department of Biomedical Sciences, Humanitas University, Milan, Italy; ^13^ IRCCS Humanitas Research Hospital, Milan, Italy; ^14^ Section on Endocrinology and Genetics, Eunice Kennedy Shriver National Institute of Child Health and Human Development (NICHD), National Institutes of Health (NIH), Bethesda, MD, United States; ^15^ Human Genetics and Precision Medicine, Institute of Molecular Biology and Biotechnology (IMBB), Foundation for Research and Technology Hellas, Heraklion, Greece; ^16^ Research Institute, ELPEN, Athens, Greece; ^17^ Department of Genetics and Molecular Pathology, Centre for Cancer Biology, An SA Pathology and University of South Australia Alliance, Adelaide, SA, Australia

**Keywords:** peptidylglycine α-amidating monooxygenase, whole exome sequencing, Cushing’s disease, prolactinoma, thyrotrophinoma, pituitary adenomas

## Abstract

**Introduction:**

Germline loss-of-function variants in *PAM*, encoding peptidylglycine α-amidating monooxygenase (PAM), were recently discovered to be enriched in conditions of pathological pituitary hypersecretion, specifically: somatotrophinoma, corticotrophinoma, and prolactinoma. PAM is the sole enzyme responsible for C-terminal amidation of peptides, and plays a role in the biosynthesis and regulation of multiple hormones, including proopiomelanocortin (POMC).

**Methods:**

We performed exome sequencing of germline and tumour DNA from 29 individuals with functioning pituitary adenomas (12 prolactinomas, 10 thyrotrophinomas, 7 cyclical Cushing’s disease). An unfiltered analysis was undertaken of all *PAM* variants with population prevalence <5%.

**Results:**

We identified five coding, non-synonymous *PAM* variants of interest amongst seven individuals (six germline, one somatic). The five variants comprised four missense variants and one truncating variant, all heterozygous. Each variant had some evidence of pathogenicity based on population prevalence, conservation scores, *in silico* predictions and/or prior functional studies. The yield of predicted deleterious *PAM* variants was thus 7/29 (24%). The variants predominated in individuals with thyrotrophinomas (4/10, 40%) and cyclical Cushing’s disease (2/7, 29%), compared to prolactinomas (1/12, 8%).

**Conclusion:**

This is the second study to demonstrate a high yield of suspected loss-of-function, predominantly germline, *PAM* variants in individuals with pathological pituitary hypersecretion. We have extended the association with corticotrophinoma to include the specific clinical entity of cyclical Cushing’s disease and demonstrated a novel association between *PAM* variants and thyrotrophinoma. *PAM* variants might act as risk alleles for pituitary adenoma formation, with a possible genotype-phenotype relationship between truncating variants and altered temporal secretion of cortisol.

## 1 Introduction

Pituitary adenomas arise from endocrine cells of the anterior pituitary. The World Health Organization classifies pituitary adenomas, now also referred to as pituitary neuroendocrine tumours, by transcription factor expression and hormone immunohistochemistry categories that relate to the cell of origin. The major pituitary adenoma lineages are: PIT1 (lactotroph, somatotroph, and thyrotroph tumours), TPIT (corticotroph tumours), SF1 (gonadotroph tumours), and no distinct cell lineage (null-cell and plurihormonal tumours) (1).

The genes that have been hitherto implicated in pituitary tumorigenesis typically respect the cell of origin paradigm. In the germline setting, *AIP* variants and X-chromosome microduplications involving *GPR101* are strongly associated with somatotrophinomas, whilst variants in *MEN1*, *PRKAR1A* and the *SDHx* genes (*SDHA*, *SDHB*, *SDHC*, *SDHD*, *SDHAF2*) are associated with all pituitary adenoma subtypes but with a predilection for lactotroph hyperplasia/adenomas. In the somatic pituitary adenoma setting, the principal driver mutations are *GNAS* and *USP8* variants, each found in approximately half of somatototrophinomas and corticotrophinomas, respectively (2).

*PAM* is a newly implicated gene in pituitary tumorigenesis, with the princeps study by Trivellin et al. earlier this year demonstrating loss-of-function *PAM* variants across hypersecretory pituitary adenoma subtypes in patients from different centres in United States and Europe (3). Out of 299 individuals with sporadic pituitary adenomas and 18 kindreds with familial isolated pituitary adenomas, they identified seven *PAM* variants with deleterious effects on protein expression and/or function. Some variants were shared between individuals. The phenotypes of cases with these *PAM* variants comprised familial gigantism, sporadic somatotrophinoma, and paediatric corticotrophinoma. The investigators proceeded to analyse genotype/phenotype data from the UK Biobank (UKBB) and found that *PAM* variants were enriched in patients with ICD-10 codes associated with sellar lesions.

*PAM* is a 974-amino acid (longest isoform), 25-exon gene which encodes peptidylglycine α-amidating monooxygenase (PAM), a bifunctional enzyme responsible for C-terminal amidation of peptides. The resultant amidated peptides can be significantly more biologically potent than their unmodified precursors (4). Through this and other mechanisms, PAM is essential to the biosynthesis and regulated processing and secretion of multiple pituitary and hypothalamic peptide hormones in addition to many neuropeptides. PAM contains two enzymatic domains – peptidylglycine α-hydroxylating monooxygenase (PHM) and peptidyl-α-hydroxyglycine α-amidating lyase (PAL) – which act sequentially to generate C-terminal amidated peptides (3, 5). PAM expression in the pituitary gland is demonstrated in the GTEx (https://gtexportal.org) and Human Protein Atlas (https://www.proteinatlas.org) databases, and this has been confirmed by dedicated pituitary studies (3, 6). To date, the Trivellin et al. study is the only evidence of a role for *PAM* variants in pituitary tumorigenesis (3). By contrast, *PAM* variants have an established role in being associated with increased risk of type 2 diabetes mellitus (T2DM) (4).

To further investigate the relationship between *PAM* and functioning pituitary adenomas, we searched for *PAM* variants in an independent Australian cohort of individuals with prolactinoma, thyrotrophinoma or cyclical Cushing’s disease. We hypothesised that *PAM* may act as a tumour suppressor gene like other pituitary tumorigenesis genes such as *MEN1* and *AIP* (2), with a somatic second-hit involving the wild-type (WT) allele in accordance with the Knudson two-hit hypothesis (7). This cohort of patients had been recruited for whole exome sequencing (WES) of paired tumour and germline DNA because of the paucity of known driver mutations in prolactinomas, thyrotrophinomas and the specific subset of corticotrophinomas associated with cyclical Cushing’s disease.

## 2 Methods

### 2.1 Patients

The study cohort comprised 29 unrelated patients with functioning pituitary adenomas (12 prolactinomas, 10 thyrotrophinomas, 7 cyclical Cushing’s disease) that had been surgically resected at tertiary referral pituitary centres in Australia: Royal Adelaide Hospital in South Australia, Royal Melbourne Hospital in Victoria, and Royal North Shore Hospital in New South Wales. None of the study subjects were part of the original cohort published by Trivellin et al. (3). Cyclical Cushing’s disease was defined by biochemical documentation of episodic cortisol excess interspersed by periods of normal or low cortisol secretion (8). Clinical and genetic characteristics of some study participants have been previously published (9–11). Pathogenic germline variants in established pituitary predisposition genes (*AIP*, *CDKN1B*, *MEN1*, *PRKAR1A*, *SDHA*, *SDHB*, *SDHC* and *SDHD*) had been excluded in all individuals.

The study was approved by the local institutional research committees (Melbourne Health: HREC/16/MH/132; Royal Adelaide Hospital: SSA/18/CALHN/445). All participants provided written informed consent to participate in the study.

### 2.2 DNA sequencing

Germline and tumour DNA was obtained from each patient, apart from a single patient in whom tumour DNA was not available.

Fresh blood samples were obtained from each patient for extraction of germline DNA from peripheral blood leucocytes. Operative tumour specimens were retrieved for somatic DNA extraction. Tumour specimens had either been stored as fresh frozen or formalin-fixed paraffin-embedded (FFPE) tissue. Both tumour and germline DNA were extracted using commercially available kits (Qiagen and Bioline) according to manufacturer protocols. FFPE samples were deparaffinised and additional DNA repair steps were performed using uracil-N-glycosylase to enzymatically remove formalin-induced cytosine deamination artefacts.

Next generation sequencing (NGS) of germline and tumour DNA was performed using whole-exome capture (Roche NimbleGen SeqCap EZ MedExome v3.0 in 27 cases; IDT xGen Exome v2 in 2 cases) sequenced on the Illumina NextSeq or NovaSeq Sequencing System.

### 2.3 Bioinformatic analysis

Variant calling was undertaken at the Australian Cancer Research Facility (ACRF) of the Centre for Cancer Biology, SA Pathology (Adelaide, Australia). The Burrows-Wheeler Alignment tool, BWA-MEM, was used to align short reads to human reference assembly GRCh37/hg19 (version b37+decoy). Variants up to a size of approx. 50 bp were called using Genome Analysis Toolkit (GATK) HaplotypeCaller package version 3.4 (12).

Raw WES data were filtered for variants in *PAM* with minor allele frequency (MAF) below 5.0% in the general population, noting that one of the key variants of interest (p.Asp563Gly) in the Trivellin et al. study has a global MAF of 4.2% (gnomAD v4.0.0) and yet still exhibited significant reductions in both PHM and PAL activity (3).

The final set of *PAM* variants of interest was confirmed by manual inspection of raw sequencing data in Integrated Genomics Viewer (IGV). Pathogenicity predictions were made using *in silico* tools such as Combined Annotation Dependent Depletion (CADD) and Genomic Evolutionary Rate Profiling (GERP). Evolutionary conservation of the *PAM* variants was derived from protein sequence alignments of the following sequences from UniProt and Clustal Omega: human PAM-1 (P19021-5), chimpanzee PAM-1 (A0A2I3SM67-1), rat PAM-1 (P14925-1), Aplysia PAM-1 (Q9NJI4-1), Drosophila PHM (O01404-1) and PAL2 (Q9W1L5-1), and Chlamydomonas PAM (A0A0S2C767-1), as previously described (3). 3D models of WT and mutant PAM proteins were generated using HOPE, an automated program that analyses the structural and functional effects of point mutations using a range of information sources including calculations on the 3D coordinates of the protein by using WHAT IF Web services, sequence annotations from the UniProt database, predictions by DAS services, and the AlphaFold Protein Structure Database (AlphaFold DB, https://alphafold.ebi.ac.uk) (13–15).

### 2.4 Loss of heterozygosity studies

We investigated potential *PAM* somatic variants in all patients as described above. In cases with a germline *PAM* variant, we also screened for loss of heterozygosity (LOH) by comparing variant frequencies in germline *vs.* tumour DNA.

### 2.5 Review of existing patients with *PAM* variants and Cushing’s disease

To investigate a specific link between *PAM* variants and cyclical Cushing’s disease, we sought to identify cases of cyclical hypercortisolism amongst the previously described cohort published by Trivellin et al. (3). We hence examined clinical data from the subset of patients in the Trivellin et al. cohort derived from the National Institutes of Health (NIH). These patients were originally recruited by the NIH in accordance with the NIH research protocol, 97-CH-0076 (ClinicalTrials.gov: NCT00001595).

## 3 Results

### 3.1 Yield of *PAM* variants in study cohort

Amongst 29 patients with functioning pituitary adenomas, we identified 10 coding, non-synonymous single nucleotide variants in *PAM* that were uncommon (MAF <5.0%), of adequate quality, and not located in a low complexity region. Of these 10 variants, four were discarded due to low coverage (total depth <10X), and one (p.Glu491Asp) was not further studied due to a previous likely benign classification based on functional studies and other data (3).

The five remaining variants comprised four missense variants and one truncating variant as outlined in **Table 1**. The five variants of interest were found in seven individuals, all in the heterozygous state and confirmed by inspection of raw sequencing data. The variant was germline in six cases and somatic in the remaining case. Amongst the six germline cases, the variant was present in both the germline DNA and tumour DNA in five patients, whilst the tumour status was unknown in the remaining case as tumour DNA was unavailable. All five variants had a predicted or proven deleterious effect on protein function as detailed below. The final yield of uncommon, predicted deleterious *PAM* variants was thus 7/29 (24%). The variants predominated in individuals with thyrotrophinomas (4/10, 40%) and cyclical Cushing’s disease (2/7, 29%), compared to individuals with prolactinomas (1/12, 8%).

**Table 1:** Molecular characteristics of the five predicted deleterious *PAM* variants.

Variant type	gDNA position	cDNA position	AA position	PAM domain	PA type	Variant frequency	gnomAD MAF	CADD	GERP	Previously reported
missense	chr5:102282583	c.569G>A	p.Arg190His	PHMcc	PRL	germline 46%, tumour 38%	0.09%	24.8	4.56	yes: single case of pituitary hyperfunction (3)
missense	chr5:102282589	c.575C>T	p.Pro192Leu	PHMcc	TSH	germline 58%, tumour 21%	1.08%	27.8	4.48	no
truncating	chr5:102284105	c.599_600insGA	p.Tyr200Ter	PHMcc	CCD	germline 40%*	0.0007%		5.75	no
missense	chr5:102338811	c.1688A>G	p.Asp563Gly	PALcc	TSH (n=2), CCD	TSH 1: germline 55%, tumour 27%; TSH 2: germline 57%, tumour 48%; CCD: germline 50%, tumour 48%	4.22%	31	5.9	yes: multiple PA and T2DM cases, demonstrated reduced amidating activity (3, 4)
missense	chr5:102360927	c.2578C>T	p.Pro860Ser	C-terminus	TSH	tumour 44%**	0.03%	23.6	4.95	no

AA, amino acid; CADD, Combined Annotation Dependent Depletion score; CCD, cyclical Cushing’s disease; cDNA, complementary DNA; gDNA, genomic DNA (hg19 build); GERP, Genomic Evolutionary Rate Profiling score; gnomAD, Genome Aggregation Database (v4.0.0); MAF, minor allele frequency; PA, pituitary adenoma; PALcc, peptidyl-α-hydroxyglycine α-amidating lyase catalytic core; PHMcc, peptidylglycine α-hydroxylating monooxygenase catalytic core; PRL, prolactinoma; T2DM, type 2 diabetes mellitus; TSH, thyrotrophinoma; * tumour DNA unavailable, ** absent in germline DNA; - not found.

Characteristics of the individuals carrying the predicted deleterious *PAM* variants are provided in **Table 2**.

**Table 2:** Clinical characteristics of the seven patients with predicted deleterious *PAM* variants.

*PAM* variant	Variant location	Diagnosis	Sex	Age at diagnosis (years)	Tumour size (mm)	Local invasion	Treatment	Tumour remnant	Other notable features
c.569G>A (p.Arg190His)	germline	PRL	M	53	18	bilateral cavernous sinuses	DA, surgery, RTx	yes	prolactin 145-fold elevated at diagnosis; severe DA intolerance
c.575C>T (p.Pro192Leu)	germline	TSH	M	60	7	no	SSA, surgery	no	TSH-dependent thyrotoxicosis, no cosecretion
c.599_600insGA (p.Tyr200Ter)	germline	CCD	F	40	7	no	surgery	no	high Ki-67 (8%) in tumour, also had type 2 diabetes (resolved after hypophysectomy) and prior papillary thyroid cancer
c.1688A>G (p.Asp563Gly)	germline	TSH	F	46	15	suprasellar extension	surgery	yes	TSH-dependent thyrotoxicosis, no cosecretion
c.1688A>G (p.Asp563Gly)	germline	TSH	F	45	14	left cavernous sinus	SSA, surgery	no	TSH-dependent thyrotoxicosis, no cosecretion
c.1688A>G (p.Asp563Gly)	germline	CCD	M	47	71	sphenoid and bilateral cavernous sinuses, skull base	surgery, DA	yes	intermittent hypercortisolism: 24 hr urine free cortisol up to 35-fold elevated, no clinical features of Cushing’s syndrome
c.2578C>T (p.Pro860Ser)	somatic	TSH	F	60	21	bilateral cavernous sinuses	CBZ, surgery	yes	TSH-dependent thyrotoxicosis, no cosecretion

CBZ, carbimazole; CCD, cyclical Cushing’s disease; DA, dopamine agonist; F, female; M, male; PRL, prolactinoma; RTx, radiotherapy; SSA, somatostatin analogue; TSH, thyrotrophinoma; TSS, transsphenoidal surgery.

### 3.2 Variant analysis

As shown in **Table 1**, all five *PAM* variants of interest had high GERP scores (>2). The four missense variants additionally had high CADD scores (>20). The truncating variant (p.Tyr200Ter) is a 2 bp frameshift insertion which is predicted to introduce a premature stop codon immediately at the same position and is likely to result in nonsense mediated decay as it is situated in exon 8.

**Figure 1:**
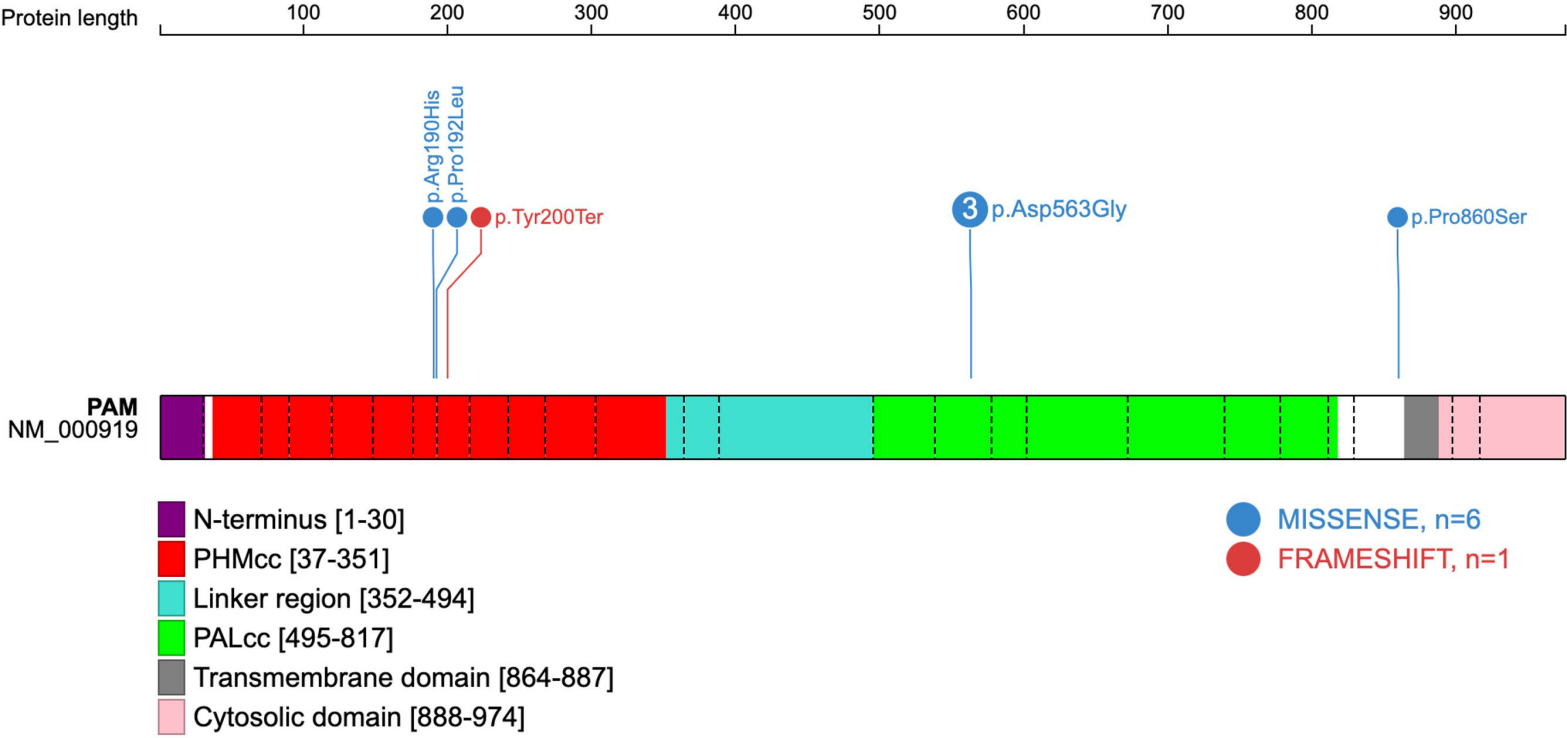
Schematic representation of the human *PAM* protein (PAM-1 isoform NM_000919.3). The domains have been previously deduced, and the schematic gene representation has been adapted from reference (3), with inclusion of the five predicted deleterious variants found amongst seven patients of the present cohort. Exon boundaries are indicated by dotted lines and codon numbers are shown. PHMcc, catalytic core of PHM; PALcc, catalytic core of PAL.

Four of the five variants correspond to the critical catalytic domains of PAM as depicted in **Figure 1**. The p.Arg190His PHMcc variant has previously been reported in association with unspecified pituitary hyperfunction in the UKBB (3). The nearby p.Pro192Leu variant, also situated in the PHMcc, is located at the same residue as a variant of interest (p.Pro192Arg) in a UKBB case with a diagnosis of syndrome of inappropriate secretion of antidiuretic hormone (3), but it is relatively frequent in the general population with a maximum population MAF of 1.4% in Europeans. The truncating variant (p.Tyr200Ter) is also situated in the PHMcc. The p.Asp563Gly PALcc variant was the only variant found in multiple patients. This variant is relatively frequent in the general population, and was not significantly more frequent in our cohort (global gnomAD MAF 4.2% *vs*. 5.2%, *p*-value non-significant by 2-tailed Fisher’s exact test). However, p.Asp563Gly is one of the key *PAM* risk alleles in T2DM (4), and has been demonstrated repeatedly to cause significant reductions in total amidating activity, PHM activity and PAL activity (3, 4). The remaining variant (p.Pro860Ser) is situated adjacent to the transmembrane domain in the C-terminal end of PAM. Whilst this is a non-catalytic region, a nearby variant (p.Leu856Pro) was found in association with Cushing’s disease in UKBB (3), and the juxtamembrane region encompassing these variants has been shown in a rat model to influence pH-dependent aggregation of PAM in secretory granules (16).

**Figure 2:**
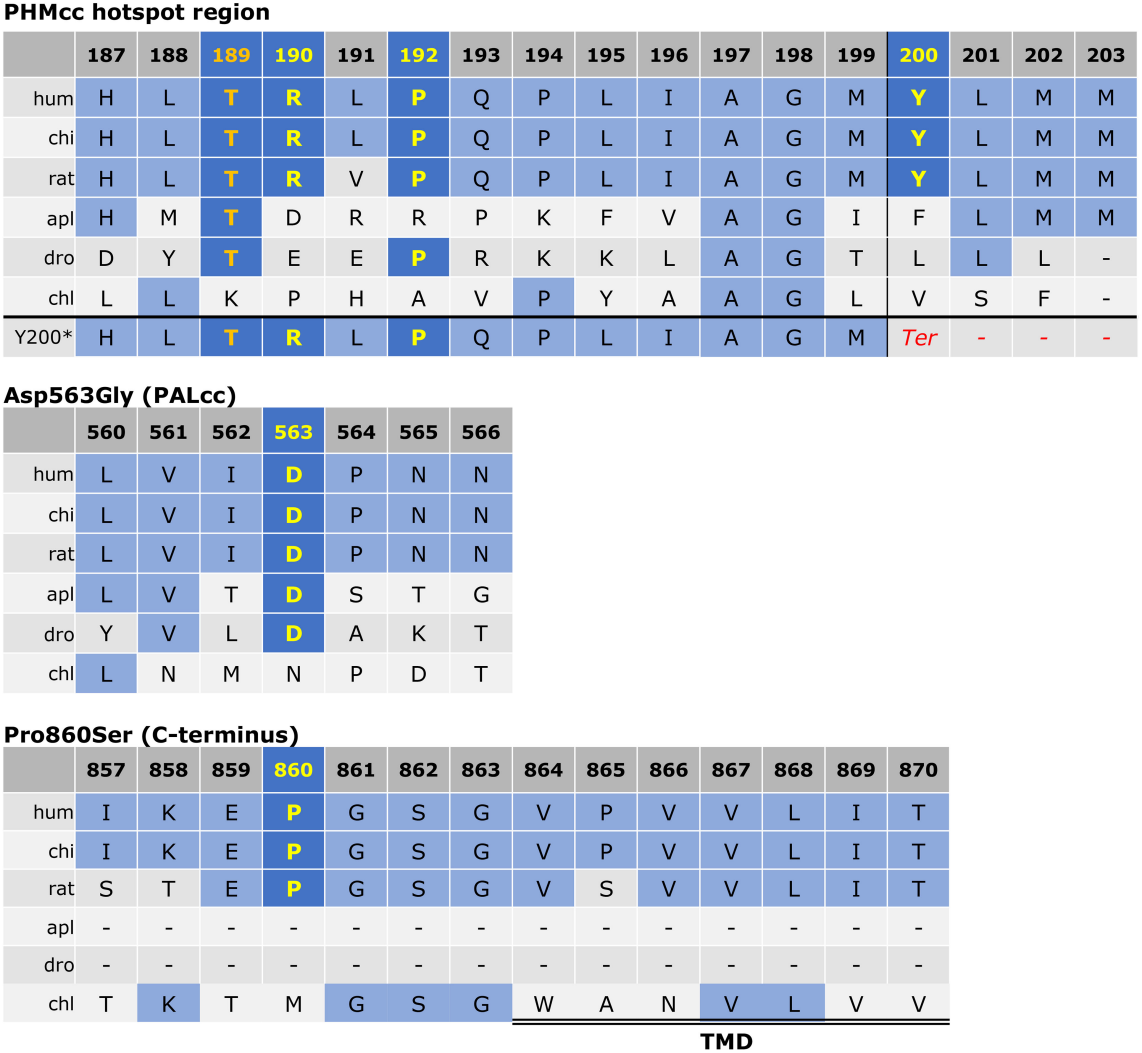
Protein sequence alignment for *PAM* variants of interest. As previously described (3), the alignment was derived via the Clustal Omega program with default settings and the UniProt alignment tool. The conserved residues of interest are shown in yellow. The Thr189 residue was used as an engineered inactive mutant by Trivellin et al. (3) and is shown in orange for reference. The other variants (p.Arg190His, p.Pro192Leu, p.Tyr200Ter, p.Asp563Gly, and p.Pro860Ser) were observed in our cohort. For the p.Tyr200Ter variant, the predicted frameshift is visible in the last row with introduction of a premature stop codon shown in red. The start of the transmembrane domain (TMD) adjacent to p.Pro860Ser is indicated by the double line. PHMcc, catalytic core of PHM; PALcc, catalytic core of PAL.

**Figure 3:**
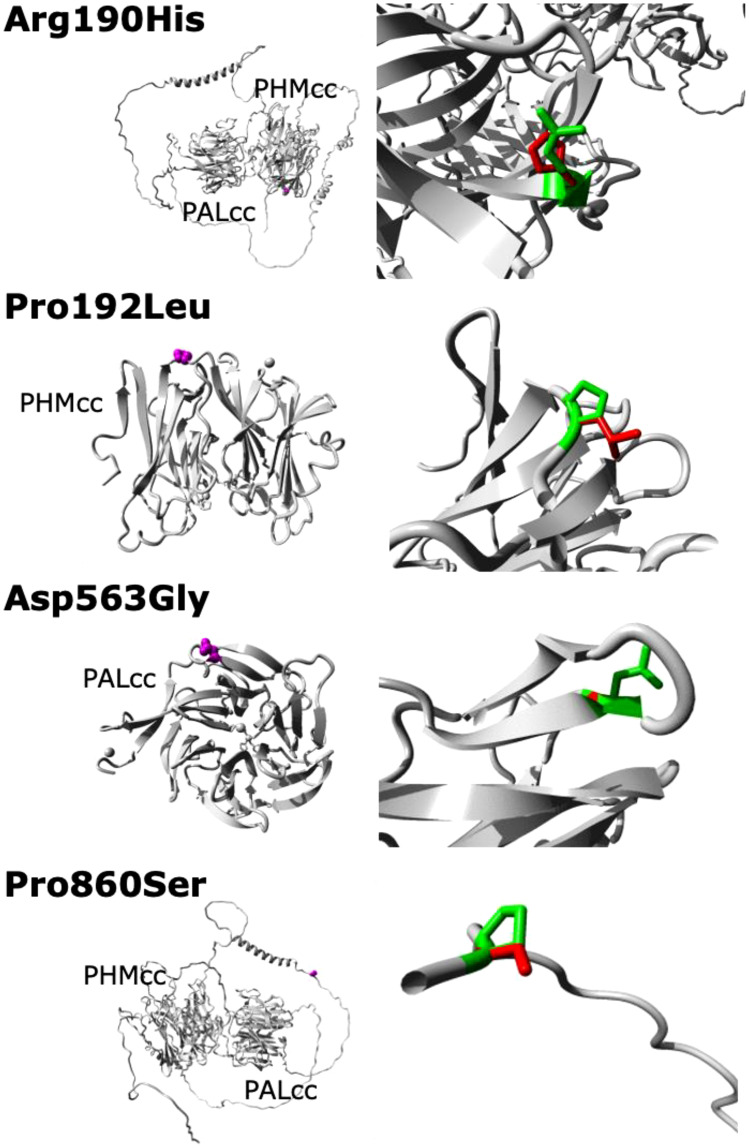
Predicted crystal structures of the WT and missense mutant PAM proteins. No structural data are available for full-length PAM; in particular, no structures are available for the linker region between PHMcc and PALcc or for the region between PALcc and the transmembrane domain (these regions are shown here as unfolded, except for two helical regions in the linker region). The left panels show an overview of the mutant proteins in ribbon-presentation, with the proteins coloured grey and the side chains of the mutated residues coloured magenta and shown as small spheres. The right panels show magnified views of the mutant resides, with the side chains of the WT and mutant residues coloured green and red, respectively. PHMcc, catalytic core of PHM; PALcc, catalytic core of PAL.

Although functional assays were not available to verify loss-of-function effects by the variants in our cohort, the missense variants were located in well-conserved positions with deleterious *in silico* predictions, and the truncating variant is a definitive null variant. Evolutionary conservation of the five *PAM* variants is demonstrated in **Figure 2**. The predicted crystal structures from the missense variants are shown in **Figure 3**.

### 3.3 Loss of heterozygosity studies

Tumour DNA was not available from the patient with the truncating *PAM* variant (cyclical Cushing’s disease). Another patient only exhibited their *PAM* variant in tumour DNA (thyrotrophinoma), whilst their germline DNA was WT at this allele. In the other five patients with predicted deleterious *PAM* variants, persistent heterozygosity of the variant of interest was observed in tumour DNA as shown in **Table 1**, thereby excluding LOH.

### 3.4 Review of existing patients with *PAM* variants and Cushing’s disease

The NIH cohort comprised patients from around the world. Inclusion in the NIH protocol required patients to have a documented period of at least 6 months of consistent hypercortisolism in order to permit the subsequent NIH diagnostic testing algorithm. Thus, cyclical hypercortisolism with cycles of less than 6 months was specifically excluded by the study design. There was accordingly no evidence of cyclicity amongst patients with *PAM* variants and Cushing’s disease in the NIH cohort.

## 4 Discussion

Germline genetic variation is traditionally considered to account for only 5% of pituitary adenomas (2). Herein we report predicted deleterious *PAM* variants in 7/29 (24%) individuals with functioning pituitary adenomas, with higher rates in the setting of thyrotrophinoma (4/10, 40%) and cyclical Cushing’s disease (2/7, 29%) compared to prolactinoma (1/12, 8%). The five observed *PAM* variants each exhibit some evidence of pathogenicity based on population prevalence, conservation scores, *in silico* predictions and/or prior functional studies. Our findings corroborate the seminal study by Trivellin et al. linking deleterious *PAM* variants with a variety of functioning pituitary adenomas, namely: somatotrophinoma, paediatric corticotrophinoma, and prolactinoma (3). We now demonstrate the first cases of *PAM* variants associated with cyclical Cushing’s disease and thyrotrophinomas. The discovery of *PAM* as a pituitary tumorigenesis gene is striking in its strong relationship with a mixture of pituitary adenoma subtypes across PIT1 and TPIT lineages.

Although the relationship between *PAM* and pituitary tumorigenesis is new, PAM has been intimately associated with pituitary function since discovery of the protein in 1982 as the porcine pituitary enzyme responsible for C-terminal amide formation and hence its critical involvement in the production of α-MSH from POMC (17). PAM is now known to be essential for the generation of all other amidated peptides, such as oxytocin and vasopressin, and other processes, including secretory granule biogenesis and retrograde signalling from the granule lumen to nucleus (5). C-terminal amidation is achieved by sequential activity of the two catalytic domains of PAM. Firstly, PHMcc functions as a monooxygenase with copper and ascorbate to α-hydroxylate the substrate (glycine-extended peptide) into an intermediate (hydroxyglycine-extended peptide). Subsequently, PALcc functions as a lyase to cleave the N-C bond of the intermediate and produce the final amidated product (5, 18). Supporting a mechanistic role for *PAM* variants in pituitary tumorigenesis, several rare variants were identified in the PHMcc and PALcc regions in our study and in the Trivellin et al. study cohort and their subsequent UKBB analysis (3). In the T2DM setting, the key *PAM* risk alleles (rs78408340, p.Ser539Trp; rs35658696, p.Asp563Gly) are also situated in the PALcc (4).

There appears to be a particular mutational hotspot within PHMcc involving the Arg190 and Pro192 residues that were mutated in our cohort and in the Trivellin et al. study, and the Thr189 residue which was used as an engineered inactive mutant by Trivellin et al. (6% PHM activity induced by Thr189Ile compared to WT) (3). This may be explained by the role of this region in copper reduction. This region of PHMcc (Arg190-Pro194) is the only direct connection of the N-terminus region of PHMcc to the C-terminus region of PHMcc. Communication between the N- and C-terminii of PHMcc is critical in allowing the transfer of copper between the two sites, which is in turn required for substrate hydroxylation (19). Hence, variants in this region might be expected to hinder essential conformational transitions and disrupt protein function.

Our finding of a *PAM* truncating variant (p.Tyr200Ter) in the setting of cyclical Cushing’s disease is particularly noteworthy as this is only the second truncating *PAM* variant to be associated with pituitary hypersecretion (3). Apart from *PRKAR1A* variants in the subset of cyclical Cushing’s syndrome related to primary pigmented nodular adrenocortical disease (20) and a case study raising a putative link between *AHR* and cyclical Cushing’s disease (9), there are currently no genes implicated in cyclical Cushing’s syndrome. The null nature of the *PAM* truncating variant in our patient with cyclical pituitary Cushing’s, and the existing data supporting a role between *PAM* variants and pituitary tumorigenesis, together favour a true mechanistic association between cyclical Cushing’s disease and the variant. In addition, another patient in our cohort with cyclical Cushing’s disease harboured the established loss-of-function Asp563Gly variant.

The single truncating *PAM* variant (p.His778fs) in the original study by Trivellin et al. also had Cushing’s disease (3). In light of our findings, this case and other NIH-recruited cases of Cushing’s disease with *PAM* variants in the original study were reviewed by the authors for hypercortisolism cyclicity. As patients in the NIH cohort were required to have a 6-month period of sustained hypercortisolism, none of the NIH cases had cyclical Cushing’s disease with cycles of less than 6 months duration, although we cannot exclude the possibility of cycles longer than 6 months duration. In any case, the carrier parent of the child with Cushing’s disease and the p.His778fs variant was found to have abnormal circadian rhythm of cortisol secretion with a high midnight serum cortisol despite being clinically unaffected (3).

How PAM variants might lead to cyclical Cushing’s disease and altered cortisol diurnal rhythm is yet to be elucidated. Since PAM is involved in the downstream amidation of ACTH leading to α-MSH synthesis (5, 17), and α-MSH exerts negative feedback on corticotrophin-releasing hormone (CRH) in rodents (21, 22), PAM could be considered to be an indirect negative regulator of ACTH activity. In addition, PAM might alter the timing of negative feedback on ACTH through its roles in secretory granule biogenesis and communication between secretory granules and the nucleus. Temporal disturbances in the hypothalamic-pituitary-adrenal (HPA) axis might also be an indirect consequence of the effect of PAM on a host of other amidated neuropeptides functioning in the hypothalamus and throughout the body, with synthesis of such amidated peptides expected to be reduced by deleterious *PAM* variants. Supporting the notion of PAM-mediated restraint of ACTH excess, there is a tendency towards greater *PAM* mRNA expression in silent corticotrophinomas compared to functioning corticotrophinomas (23). Taken together, PAM appears to be an indirect negative regulator of ACTH, and mutant PAM may thus lead to ACTH excess, but it remains unclear how mutant PAM might cause periodic disturbances in ACTH production, which is what would be expected if it were the underlying cause of cyclical hypercortisolism and abnormal cortisol diurnal rhythm. Future studies looking into whether PAM expression is light-entrained and exploring the temporal relationships between PAM and ACTH expression may help determine if *PAM* is a clock gene capable of influencing circadian and infradian ACTH variations.

Our study also demonstrates a novel association between *PAM* variants and thyrotrophinoma, with *PAM* variants found in 4/10 thyrotrophinoma patients. The variant was germline in three cases, with two of these cases harbouring the established loss-of-function p.Asp563Gly variant and the other harbouring the p.Pro192Leu variant situated in the PHMcc with high scores by CADD (27.8) and GERP (4.48) predicting a deleterious effect. The remaining variant was purely somatic, situated in the C-terminus and with a slightly lower CADD score of 23.6. If the latter variant is disregarded, the finding of *PAM* variants in 30% of thyrotrophinoma cases remains striking given the rarity of thyrotrophinomas and the lack of any known driver mutations for this pituitary adenoma subtype in the few genomic studies that have included thyrotrophinomas (24, 25).

The relatively high MAFs of some of the *PAM* variants in the pituitary adenoma setting coupled with the predicted or proven deleterious nature of some of these variants, suggest that *PAM* variants might act as pituitary adenoma risk alleles (26). This would be in keeping with the negative family histories in most pituitary adenoma cases carrying germline *PAM* variants (3). *PAM* variants have similarly been found to act as risk alleles for T2DM rather than causing monogenic diabetes (4). As pituitary tumours in general are highly prevalent (10% detection rate on MRI of healthy controls (27)) and infrequently familial (5% of all pituitary adenoma cases (2)), it is plausible that genetic predisposition to the condition relates more commonly to risk alleles than highly penetrant monogenic conditions. Even in the case of *AIP* variants, which are the most prevalent cause of familial pituitary tumours, the penetrance of pituitary adenomas is only 15-30% (2). Whether there are genotype-phenotype correlations amongst *PAM* variants in the pituitary setting remains to be seen. Truncating *PAM* variants may be specifically associated with altered temporal secretion of cortisol given that the only two reported truncating variants have been associated with loss of diurnal rhythm of cortisol secretion (3) and now with cyclical Cushing’s disease.

As in the study by Trivellin et al. (3), we did not observe LOH in the tumours of our patients with germline *PAM* variants. This may be explained by somatic second-hits that are not identifiable within the limits of the genetic testing methodologies used, such that *PAM* may still act as a tumour suppressor like other genes involved in pituitary tumorigenesis, such as *MEN1* and *AIP* (2). Alternatively, *PAM* variants may cause pituitary tumorigenesis through haploinsufficiency. There are animal studies demonstrating the effects of *PAM* haploinsufficiency, with *PAM* ^+/-^ mice demonstrating altered levels of several amidated peptides (18).

The predicted deleterious *PAM* variants found in this Australian cohort complement the findings from the original cohort of patients from the Unites States and Europe (3). Independently demonstrating a relationship between gene variants and a disease in different cohorts is a critical component of the ClinGen Clinical Validity Classification system (28). These data may thus help confirm the gene-disease relationship between *PAM* and functioning pituitary adenomas. There are, however, limitations in our study. The study was initially designed to search for driver mutations and hence focused on sequencing of tumour-germline DNA pairs from individuals, without the additional ethical approval needed to sequence and phenotype family members which might have otherwise helped elucidate the role of the germline *PAM* variants found here. In addition, our study was relatively small, and employed only WES, which will identify most sequence variants and some copy number variation but miss other important genetic aberrations. Finally, although we have depicted the expected crystal structure changes induced by the missense *PAM* variants identified in our cohort, these predictions are based on incomplete data. Structural data do not exist for full-length PAM; in particular, we have had to include low probability data for the linker and cytosolic regions of PAM.

We anticipate that further independent pituitary adenoma cohorts will be examined for *PAM* variants, which will help to confirm the proposed gene-disease relationship, and hopefully solve unanswered questions, such as whether *PAM* is a traditional tumour suppressor or rather an haploinsufficient gene, and precisely how PAM inactivation results in pituitary hormone hypersecretion. Future directions of research should include *PAM* sequencing in independent cohorts of thyrotrophinomas, and further exploration of the relationship between PAM and POMC, including temporal effects, to determine how *PAM* variants might produce cyclical Cushing’s disease and abnormal cortisol diurnal rhythm.

## Data availability statement

The datasets presented in this article are not readily available because of patient confidentiality. Requests to access the datasets should be directed to A/Prof Sunita De Sousa.

## Ethics statement

The studies involving humans were approved by Melbourne Health: HREC/16/MH/132; Royal Adelaide Hospital: SSA/18/CALHN/445. The studies were conducted in accordance with the local legislation and institutional requirements. The participants provided their written informed consent to participate in this study.

## Author contributions

SD: Conceptualization, Data curation, Formal Analysis, Funding acquisition, Investigation, Methodology, Project administration, Visualization, Writing – original draft, Writing – review & editing. AS: Conceptualization, Funding acquisition, Investigation, Writing – review & editing. CY: Conceptualization, Funding acquisition, Writing – review & editing. RC-B: Investigation, Writing – review & editing. SS: Investigation, Writing – review & editing. JK: Investigation, Writing – review & editing. JT: Data curation, Formal Analysis, Writing – review & editing. GT: Data curation, Writing – review & editing. AL: Writing – review & editing. CS: Data curation, Writing – review & editing. DT: Conceptualization, Investigation, Supervision, Writing – review & editing. HS: Conceptualization, Methodology, Supervision, Writing – review & editing.
